# Lifespan Extension of *Podospora anserina* Mic60-Subcomplex Mutants Depends on Cardiolipin Remodeling

**DOI:** 10.3390/ijms23094741

**Published:** 2022-04-25

**Authors:** Lisa-Marie Marschall, Verena Warnsmann, Anja C. Meeßen, Timo Löser, Heinz D. Osiewacz

**Affiliations:** Institute of Molecular Biosciences, Faculty of Biosciences, Goethe-University, Max-von-Laue-Str. 9, 60438 Frankfurt, Germany; l.marschall@bio.uni-frankfurt.de (L.-M.M.); warnsmann@bio.uni-frankfurt.de (V.W.); meessen@bio.uni-frankfurt.de (A.C.M.); loeserti@uni-mainz.de (T.L.)

**Keywords:** *Podospora anserina*, aging, mitochondria, cristae, MICOS, cardiolipin, tafazzin

## Abstract

Function of mitochondria largely depends on a characteristic ultrastructure with typical invaginations, namely the cristae of the inner mitochondrial membrane. The mitochondrial signature phospholipid cardiolipin (CL), the F_1_F_o_-ATP-synthase, and the ‘mitochondrial contact site and cristae organizing system’ (MICOS) complex are involved in this process. Previous studies with *Podospora anserina* demonstrated that manipulation of MICOS leads to altered cristae structure and prolongs lifespan. While longevity of Mic10-subcomplex mutants is induced by mitohormesis, the underlying mechanism in the Mic60-subcomplex deletion mutants was unclear. Since several studies indicated a connection between MICOS and phospholipid composition, we now analyzed the impact of MICOS on mitochondrial phospholipid metabolism. Data from lipidomic analysis identified alterations in phospholipid profile and acyl composition of CL in Mic60-subcomplex mutants. These changes appear to have beneficial effects on membrane properties and promote longevity. Impairments of CL remodeling in a PaMIC60 ablated mutant lead to a complete abrogation of longevity. This effect is reversed by supplementation of the growth medium with linoleic acid, a fatty acid which allows the formation of tetra-octadecanoyl CL. In the *PaMic60* deletion mutant, this CL species appears to lead to longevity. Overall, our data demonstrate a tight connection between MICOS, the regulation of mitochondrial phospholipid homeostasis, and aging of *P. anserina*.

## 1. Introduction

Aging of biological systems is a complex process leading to irreversible time-dependent decrease of physiological functions and an increase in morbidity and mortality. The process is under the control of environmental, stochastic, and genetic traits. There are a number of genetically controlled pathways influencing aging and lifespan [1,2,3,4]. Pathways involved in keeping a healthy population of mitochondria, the cellular power plants with many essential functions, have been demonstrated to play a key role in aging from yeast to humans [5,6,7,8,9,10,11,12,13].

*Podospora anserina* is a filamentous fungus with a strong mitochondrial etiology of aging (for review, see [14,15]). Mitochondria form a filamentous network in juvenile hyphae, but during aging, this network disintegrates, resulting in fragmented mitochondria [16]. In the aging process, the ultrastructure changes from lamellar cristae to vesicular units [17,18]. A molecular model explains that the underlying processes result from the dissociation of the F_1_F_o_-ATP-synthase dimers at the cristae tips and of a protein complex at the basis of cristae, followed by the retraction of cristae and the formation of vesicles. The dissociation of F_1_F_o_-ATP-synthase dimers was followed by cryo-electron microscopy [17] and subsequently by studying genetic mutants impaired in dimerization of F_1_F_o_-ATP-synthase. Ablation of the assembly factor PaATPE leads to the loss of lamellar cristae and a mitophagy-dependent decreased lifespan [19,20]. However, the impact of the ‘mitochondrial contact site and cristae organizing system’ (MICOS) complex at the basis of cristae was only recently studied in detail [21]. We identified five homologs of the six well-known yeast MICOS subunits in *P. anserina*, also organized in two subcomplexes (Mic60-subcomplex: PaMIC60 and PaMIC19 as well as Mic10-subcomplex: PaMIC10, PaMIC26, and PaMIC12). For the first time, a link between MICOS and the aging process was demonstrated. In this study, a counterintuitive lifespan extension was found in mutants in which individual components were ablated. Moreover, it was shown that longevity of Mic10-subcomplex mutants results from mitohormesis, the beneficial effect of mild oxidative stress [21]. Until now, the underlying mechanism of longevity in deletion mutants of the Mic60-subcomplex has not been identified.

Here we report evidence demonstrating an important role of phospholipids in the control of the lifespan of the *PaMic60* deletion mutant. In this mutant, phospholipid profile and acyl composition of the mitochondrial signature phospholipid cardiolipin (CL) are altered in comparison to the wild type. Our study unraveled an essential role of CL remodeling for longevity of the *PaMic60* deletion mutant. In addition, we extended the knowledge about the relevance of mitochondrial phospholipid metabolism for aging of *P. anserina*.

## 2. Results and Discussion

### 2.1. Loss of MICOS Subunits Leads to Changes in Phospholipid Metabolism

In a recent study we identified a link between the MICOS complex and aging of *P. anserina*. We found that loss of each of the two MICOS subcomplexes leads to unexpected longevity. While loss of Mic10-subcomplex results in mitohormesis-induced lifespan extension, the underlying mechanism leading to longevity in Mic60-subcomplex mutants appears to be caused by a different mechanism which may be linked to phospholipid metabolism [21]. This possibility is supported by the demonstration of the interaction between human MIC60 and components of the cardiolipin synthesis pathway, including phosphatidylglycerophosphate synthase (PGS1) and cardiolipin synthase (CRD1) [22]. Moreover, in several organisms, CL is known to stabilize the F_1_F_o_-ATP-synthase as well as the MICOS complex [23,24,25], which is localized at the crista junctions and involved in cristae formation [26,27,28].

**Figure 1:**
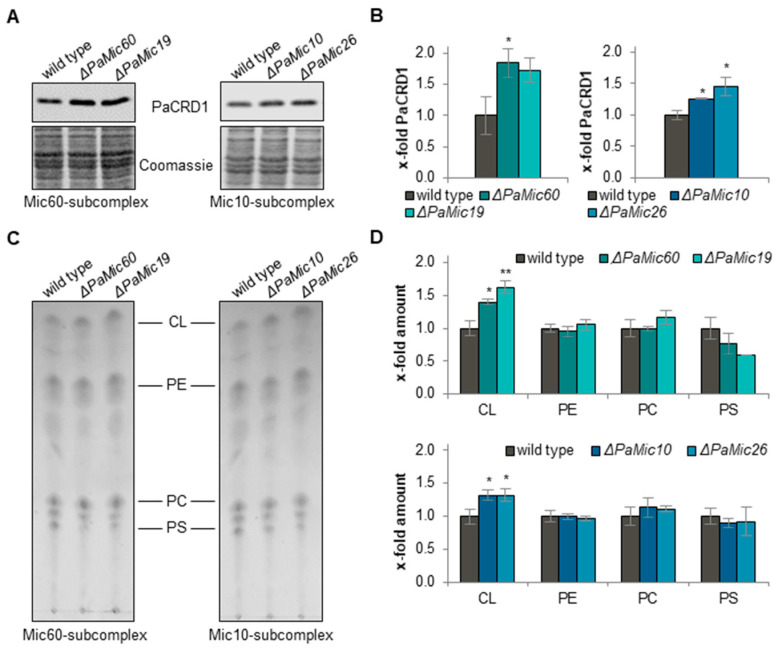
Loss of MICOS impacts cardiolipin synthesis. (**A**) Representative western blot analysis of mitochondrial protein extracts of *P. anserina* wild type, Δ*PaMic60*, Δ*PaMic19*, Δ*PaMic10*, Δ*PaMic26* using a PaCRD1 antibody. (**B**) Quantification of PaCRD1 amount normalized to the Coomassie-stained gel. PaCRD1 amount in wild type cultures was set to 1. Data represent mean ± SD (3 biological replicates each). (**C**) One-dimensional thin layer chromatography analyses of mitochondrial phospholipids of the wild type, Δ*PaMic60*, Δ*PaMic19*, Δ*PaMic10* and Δ*PaMic26*. CL: cardiolipin; PE: phosphatidylethanolamine; PC: phosphatidylcholine; PS: phosphatidylserine. (**D**) Quantification of phospholipid spots in (**C**). Intensity of each spot was determined and related to the intensity of the whole track. Phospholipid values of the wild type were set to 1. Data represent mean ± SD (3 biological replicates each). * *p* < 0.05, ** *p* < 0.01.

In order to investigate the potential impact of MICOS subunits on phospholipid metabolism, we first analyzed the amount of PaCRD1 in isolated mitochondria of the *P. anserina* wild type and MICOS deletion mutants. Loss of Mic60-subcomplex subunits, PaMIC60 or PaMIC19, led to an approximately 1.8-fold increase of PaCRD1 abundance (Figure 1A,B). Similarly, in the Mic10-subcomplex mutants, we observed a slight 1.3-fold increase in the *PaMic10* deletion mutant and a 1.4-fold increase in the Δ*PaMic26* mutant, respectively.

To determine whether the observed changes lead to altered CL levels, we performed a thin layer chromatography analysis. In the deletion strains of both subcomplexes, the altered PaCRD1 level was associated with an increase of CL (Figure 1C,D). The absence of PaMIC60 or PaMIC19 resulted in an increase in CL abundance of approximately 1.5-fold. Loss of Mic10-subcomplex subunits PaMIC10 or PaMIC26 led to a 1.3-fold increase of CL level. The abundance of other phospholipids such as phosphatidylethanolamine (PE), phosphatidylcholine (PC), and phosphatidylserine (PS) was hardly altered. A link between altered phospholipid composition and extended lifespan was already described in another *P. anserina* mutant (Δ*PaIap*) [29]. It appears that the observed significant changes in CL levels of Δ*PaMic60* and Δ*PaMic19* mutant may contribute to longevity of the Mic60-subcomplex deletion mutants.

**Figure 2:**
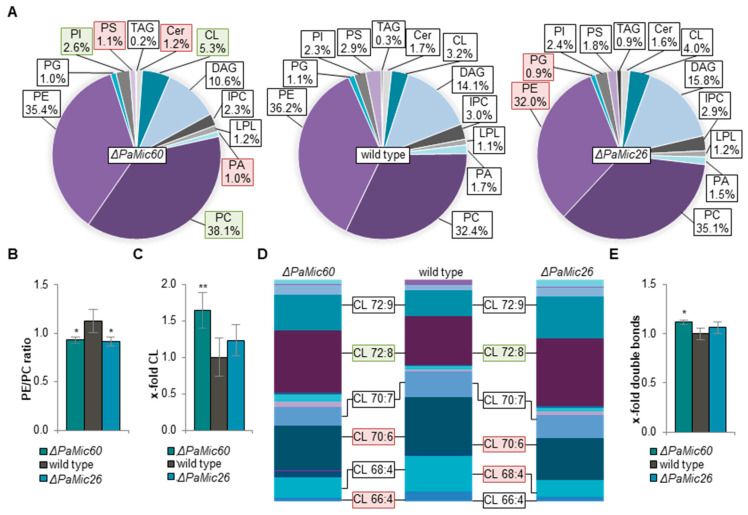
Ablation of MICOS subunits alters mitochondrial phospholipid composition. (**A**) Lipid profile of mitochondrial protein extracts of *P. anserina* wild type, Δ*PaMic60* and Δ*PaMic26*. Significant differences to the wild type are marked by green (increase) or red (decrease) boxes, respectively. Exact values are shown in Supplementary Materials (Table S1). TAG: triacylglycerol; Cer: ceramide; CL: cardiolipin; DAG: diacylglycerol; IPC: inositol phosphorylceramide; LPL: lyso-phospholipids; PA: phosphatidic acid; PC: phosphatidylcholine; PE: phosphatidylethanolamine; PG: phosphatidylglycerol; PI: phosphatidylinositol; PS: phosphatidylserine. (**B**) PE/PC ratio of wild type, Δ*PaMic60*, and Δ*PaMic26*. (**C**) Comparative analysis of CL from (**A**). Total amount of CL in wild-type cultures was set to 1. (**D**) Graphical illustration of different CL species in wild type, Δ*PaMic60*, and Δ*PaMic26* according to total length of all four acyl chains (66–74) and total degree of unsaturation (4–11). The most abundant CL species are represented. Significant differences to wild type of increased or reduced CL species are marked by green or red boxes, respectively. Exact values are shown in Supplementary Materials (Table S2). (**E**) Total double bonds across all lipids. Total amount of double bonds in wild type was set to 1. Exact values are shown in Supplementary Materials (Table S3A). Data represent mean ± SD (5 biological replicates each). * *p* < 0.05, ** *p* < 0.01.

Since thin layer chromatography allows only a very rough estimation of phospholipid classes without any information about the exact acyl composition, we set out to elucidate the impact of mitochondrial phospholipid composition and the role of CL in MICOS mutants by shotgun lipidomic analysis, which was performed by Lipotype (Dresden, Germany). For this analysis, we isolated and purified mitochondria from 6-day-old *P. anserina* cultures of the wild type, Δ*PaMic60*, and Δ*PaMic26*, respectively. Interestingly, the phospholipid composition was differentially affected in the two MICOS subcomplex mutants (Figure 2A). Compared to the wild type, the phospholipid profile of the *PaMic26* deletion strain was only marginally changed. We found a significant reduction of PE and phosphatidylglycerol (PG) as well as a slightly enhanced level of CL. In contrast, ablation of PaMIC60 had a strong effect on phospholipid composition compared to the wild type. There was a slight decrease of the precursor lipid diacylglycerol (DAG), as well as a significant reduction of ceramide, phosphatidic acid (PA), and PS. Since the latter two phospholipids are shuttled between the endoplasmic reticulum (ER) and the inner mitochondrial membrane, their decrease might indicate a reduction in ER/mitochondria contact. The MICOS complex ensures close apposition of mitochondrial membranes and thereby assists phospholipid synthesis in the outer mitochondrial membrane [30]. Therefore, it is possible that ablation of the Mic60-subcomplex impairs the exchange of specific phospholipids between mitochondria and the ER. Beside elevated phosphatidylinositol (PI) levels, a significant increase of approximately 20% of PC (32.4% to 38.1%) and 65% of CL (3.2% to 5.3%) was observed (Figure 2A).

Loss of PaMIC60 led to enhanced PC levels, while ablation of PaMIC26 resulted in decreased PE levels. In both cases, these changes resulted in a slight but significant shift of the PE/PC ratio from 0.9 to 1.1 compared to the wild type (Figure 2B). PE is a cone-shaped phospholipid inducing negative membrane curvature, and it contributes to elevated membrane stiffening [31,32]. As a counterpart, the cylindrical bilayer-forming PC increases membrane fluidity. Thus, the PE/PC ratio impacts membrane properties and therefore needs to be tightly controlled [33]. In mice, increased PE/PC ratio was shown to cause loss of membrane integrity, leading to liver failure [34]. Furthermore, studies with mammalian cells demonstrated that changes in PE/PC ratio have a dramatic effect on mitochondrial dynamics, morphology, and ultrastructure. Loss of PE results in fragmented abnormally swollen mitochondria lacking distinct cristae [35].

In addition to PC and PE, also CL has a major impact on membrane properties due to its high content of unsaturated fatty acids and its specific cone-shaped structure. There was a 1.7-fold increase of CL in the *PaMic60* deletion strain (Figure 2C). Especially for CL, it has been shown that acyl composition plays a crucial role for survival of *P. anserina* [29]. Therefore, we analyzed the different species of CL in more detail regarding length and degree of saturation of the attached acyl groups. Loss of MICOS resulted in a slightly altered distribution of CL species compared to the wild type (Figure 2D). Particularly, ablation of both PaMIC60 and PaMIC26 led to a shift from shorter-chained (e.g., CL 66:4, 68:4, 70:6) to rather long-chained acyl groups containing tetra-octadecanoyl residues (CL 72:X). The *PaMic60* and *PaMic26* deletion mutants both showed a significant increase of tetra-linoleoyl-CL (CL 4 × 18:2 or CL 72:8). In mammalian cardiac mitochondria with high respirational activity, CL 72:8 was found to be the dominant CL species [36,37]. Moreover, an increased abundance of CL 72:8 enhances mitochondrial function in mammalian models upon heart failure [38,39]. In human cells, linoleic acid is preferentially incorporated into CL, resulting in an increased amount of CL 72:8 and promoting CI activity [40]. Thus, it seems that CL 72:8 exerts a beneficial effect on mitochondrial function. Such an effect was also previously observed in a study with *P. anserina* [29]. Due to the altered phospholipid profile and distribution of CL species, the amount of double bonds in the Δ*PaMic60* mutant was slightly but significantly increased across all phospholipids reflected by more unsaturated acyl residues (Figure 2E). Such a moderate higher degree of unsaturation can positively affect the fluidity of membranes [41]. Altogether, our data suggest that in both analyzed MICOS subcomplex mutants, membrane fluidity was increased.

### 2.2. Cardiolipin Remodeling Is Necessary for Lifespan Extension of ΔPaMic60

**Figure 3:**
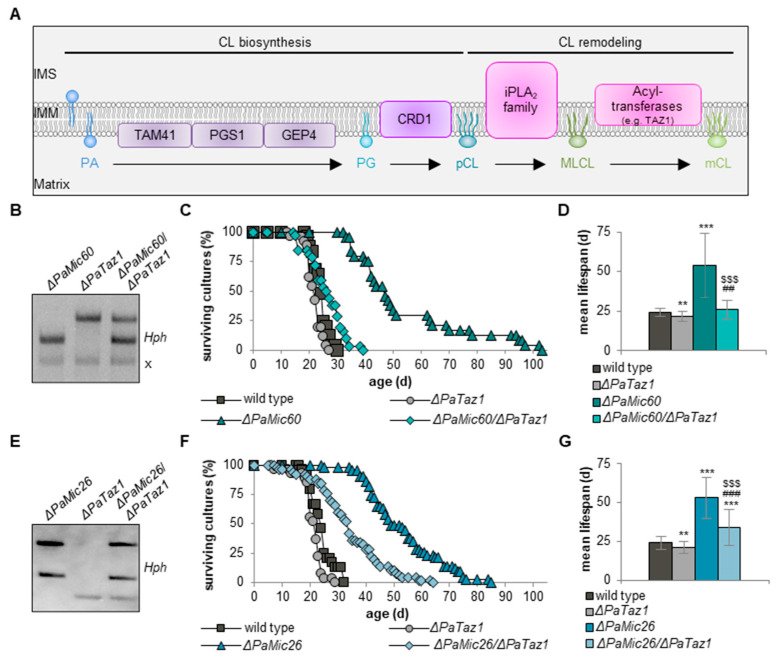
Ablation of PaTAZ1 affects longevity of MICOS mutants. (**A**) Scheme depicting components of CL biosynthesis and remodeling in the inner mitochondrial membrane. IMS: intermembrane space; IMM: inner mitochondrial membrane; PA: phosphatidic acid; TAM41: PA cytidylyltransferase; PGS1: phosphatidylglycerophosphate synthase; GEP4: phosphatidylglycerophosphatase; PG: phosphatidylglycerol; CRD1: cardiolipin synthase; pCL: premature cardiolipin; iPLA_2_ family: several phospholipases; MLCL: monolysocardiolipin; TAZ1: tafazzin; mCL: mature cardiolipin. A round head group indicates one phosphate (e.g., PA, PG), and an oval head group indicates two phosphates (e.g., pCL, MLCL, mCL). (**B**) Southern blot analysis with BamHI-digested DNA to verify the newly generated double mutant Δ*PaMic60*/Δ*PaTaz1* by the use of the hygromycin (*Hph*) resistance gene. A cross (x) marks an unspecific signal. (**C**) Survival curves of *P. anserina* wild type (*n* = 25), Δ*PaTaz1* (*n* = 26), Δ*PaMic60* (*n* = 24), and Δ*PaMic60*/Δ*PaTaz1* (*n* = 32) grown on M2 medium. (**D**) Mean lifespan of cultures from (**C**). Data represent mean ± SD. (**E**) Southern blot analysis with EcoRV-digested DNA to verify the newly generated double mutant Δ*PaMic26*/Δ*PaTaz1* by the use of the hygromycin (*Hph*) resistance gene. (**F**) Survival curves of *P. anserina* wild type (*n* = 24), Δ*PaTaz1* (*n* = 27), Δ*PaMic26* (*n* = 43), and Δ*PaMic26*/Δ*PaTaz1* (*n* = 50) grown on M2 medium. (**G**) Mean lifespan of cultures from (**F**). Data represent mean ± SD. Significant differences to wild type: ** *p* < 0.01, *** *p* < 0.001; significant differences to Δ*PaTaz1*: ^##^ *p* < 0.01, ^###^ *p* < 0.001; significant differences to Δ*PaMic60* or Δ*PaMic26*: ^$$$^ *p* < 0.001.

A common feature of the two mutants investigated in this study is the upregulation of CL synthesis. Therefore, we analyzed the role of CL synthesis and remodeling in more detail. CL biosynthesis is a process involving several intermediate steps and various enzymes (Figure 3A; reviewed in [42]). CRD1 converts PG into premature CL (pCL). Subsequently, during CL remodeling, pCL is converted into monolysocardiolipin (MLCL) by several phospholipases of the calcium-independent phospholipase A_2_ (iPLA_2_) family, followed by the generation of mature CL (mCL) by acyltransferases, such as the transacylase tafazzin (TAZ1) [43,44]. Mitochondria of a yeast *Taz1* deletion mutant still possess pCL but no mCL [45].

Previous studies revealed that loss of CRD1, which completely blocks formation of all CL species, leads to a reduced lifespan of *Drosophila melanogaster* and *P. anserina* [23,29]. To specifically address the question how MICOS ablation and subsequent lifespan extension as well as CL composition depend on each other, we generated new deletion mutants for further analysis. In order to examine the importance of CL in Δ*PaMic60*, we generated a Δ*PaMic60*/Δ*PaCrd1* double deletion mutant (Figure S1A). The generation of Δ*PaMic60*/Δ*PaCrd1* was difficult because spores from this strain germinate poorly due to a germination defect (Figure S1B). After initial formation of germination tubes on spore germination medium, growth of the double mutant completely stopped. Only after transfer of the poorly germinated spores to M2 medium and subsequent incubation for at least 5 days growth resumes. This made it impossible to maintain the same growth conditions as the single mutants. Thus, further lifespan and molecular analyses were not possible, but the impairments of the Δ*PaMic60* mutant clearly demonstrated that CL plays an important role in germination.

To examine the specific role of mCL, we first analyzed a mutant in which *PaTaz1* coding for tafazzin (PaTAZ1) is deleted [29]. Compared to the wild type, the lifespan of the Δ*PaTaz1* was only slightly reduced, by approximately 10% (mean lifespan: 22 d vs. 24 d; maximal lifespan: 29 d vs. 32 d) (Figure S2A,B). This is similar to the situation in the previously analyzed *PaCrd1* deletion mutant [29]. The lifespans of both mutants are slightly shortened, but the general loss of CL results in a stronger lifespan reduction, whereas the effect on lifespan resulting from the loss of mCL in the *PaTaz1* deletion mutant is less pronounced. More severe effects of the deletion of *Crd1* or *Taz1* have been reported in yeast. Here, deletion of the two genes was found to effect growth as well as dramatically decrease chronological lifespan [46,47].

Next, we generated a double deletion mutant lacking PaMIC60 and PaTAZ1 (Figure 3B) and analyzed the lifespan of Δ*PaMic60*/Δ*PaTaz1* (Figure 3C). Interestingly, longevity of the single Δ*PaMic60* mutant was abolished in the Δ*PaMic60*/Δ*PaTaz1* double mutant. The simultaneous ablation of PaMIC60 and PaTAZ1 caused a 70% reduced maximal lifespan (29 d vs. 103 d) and a 50% shortened mean lifespan (26 d vs. 49 d) compared to Δ*PaMic60*, leading to a wild type-like lifespan (Figure 3C,D). Obviously, longevity of the *PaMic60* deletion mutant depends on the presence of PaTAZ1.

To determine whether mCL also affects lifespan of Mic10-subcomplex mutants, we generated a double deletion mutant lacking PaMIC26 as well as PaTAZ1 (Figure 3E). Lifespan analyses revealed that the mean lifespan of Δ*PaMic26*/Δ*PaTaz1* was reduced by approximately 35% compared to Δ*PaMic26* (34 d vs. 53 d) but was still extended by around 40% compared to the wild type (34 d vs. 24 d) (Figure 3F,G). Taken together, these data demonstrate that CL remodeling is crucial for survival of the analyzed MICOS mutants. However, the importance of PaTAZ1-mediated CL remodeling seems to be different in the two MICOS subcomplex mutants. Longevity of the Mic10-subcomplex mutant only partially depends on PaTAZ1-mediated formation of mCL. In contrast, in the *PaMic60* deletion mutant, lifespan extension completely depends on PaTAZ1.

### 2.3. Ablation of PaTAZ1 Dramatically Impacts Phospholipid Metabolism

**Figure 4:**
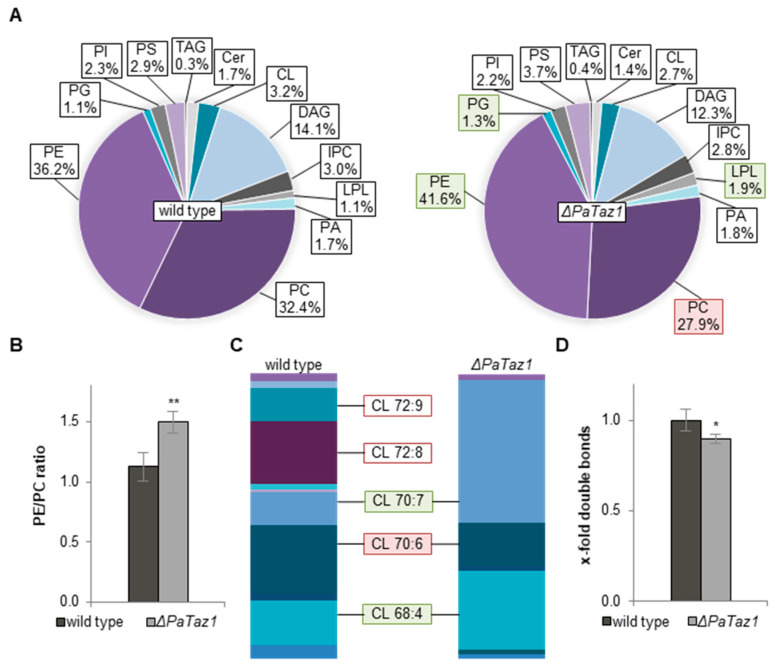
Loss of PaTAZ1 has a dramatic impact on phospholipid metabolism. (**A**) Lipid profiles of mitochondrial protein extracts of *P. anserina* wild type (*n* = 5) and Δ*PaTaz1* (*n* = 3). Significant differences to the wild type are marked with green (increase) or red (decrease) boxes, respectively. Exact values are shown in Supplementary Materials (Table S1). TAG: triacylglycerol; Cer: ceramide; CL: cardiolipin; DAG: diacylglycerol; IPC: inositol phosphorylceramide; LPL: lyso-phospholipids; PA: phosphatidic acid; PC: phosphatidylcholine; PE: phosphatidylethanolamine; PG: phosphatidylglycerol; PI: phosphatidylinositol; PS: phosphatidylserine. (**B**) PE/PC ratio of wild type and Δ*PaTaz1*. Data represent mean ± SD. (**C**) Graphical illustration of different CL species in wild type and Δ*PaTaz1* according to total length of all four acyl chains (66–72) and total degree of unsaturation (3–11). The most abundant CL species are represented (proportion > 10%). Significant differences to the wild type are marked by green (increase) or red (decrease) boxes, respectively. CL 72:8 and CL 72:9 are only represented in the wild type and are therefore marked with a red border. Exact values are shown in Supplementary Materials (Table S2). (**D**) Total double bonds across all lipids. Total amount of double bonds in wild type was set to 1. Data represent mean ± SD. Exact values are shown in Supplementary Materials (Table S3A). * *p* < 0.05, ** *p* < 0.01.

In order to elucidate the PaTAZ1-dependent CL acyl chain composition in more detail, we set out to analyze the phospholipid profile and distribution of CL species in Δ*PaTaz1*. Compared to wild type, ablation of PaTAZ1 severely impacted phospholipid composition. In addition to elevated PG and lyso-phospholipids (LPL) levels, we found a significant increase of PE by 15%. In addition, a decreased PC amount of approximately 15% was observed (Figure 4A). These changes resulted in an enhanced PE/PC ratio from 1.1 to 1.5 compared to the wild type (Figure 4B). A previous study of a *PaCrd1* deletion mutant showed a correlation between an increased PE/PC ratio and accelerated aging of *P. anserina* [29]. Similar to the Δ*PaCrd1* mutant, the lifespan of the *PaTaz1* deletion mutant was also shortened.

Next, we discriminated the different CL species regarding length and degree of saturation of attached acyl groups. Ablation of PaTAZ1 resulted in a severely altered profile of CL species, with domination of CL 68:4, CL 70:6, and CL 70:7 (Figure 4C). With a total amount of 50% of all CL species, the *PaTaz1* deletion mutant exhibited significantly increased CL 70:7 levels. Compared to the wild type, CL 68:4 was also increased by about 45%, and the amount of CL 70:6 was reduced approximately by 35%. Obviously, there were no CL 72:X species due to the loss of PaTAZ1. In particular, the complete absence of CL 72:8 in this mutant was already observed in a recent lipidomic analysis [29]. The apparent strict dependency of CL 72:X formation on the presence of PaTAZ1 differs from what is described in other organisms. In cardiac mitochondria of mice, down-regulation of TAZ1 results only in a strong reduction of CL 72:8 [48], the most common species in this tissue [49]. Nevertheless, CL 72:X species are still present in cardiac as well as in liver and kidney mitochondria of TAZ1-deficient mice [48]. The complete loss of CL 72:X and the overall altered phospholipid profile were accompanied by a significant decrease of double bonds of approximately 10% across all mitochondrial phospholipids of *P. anserina* (Figure 4D). Overall, our findings suggest that loss of PaTAZ1 dramatically impairs phospholipid metabolism and, in particular, CL remodeling, which is accompanied by a shortened lifespan in *P. anserina*. Due to the strong impact of PaTAZ1 ablation on the CL profile, the Δ*PaTaz1* mutant allowed us to uncover whether or not CL acyl chain composition plays a role in MICOS mutants.

### 2.4. Simultaneous Loss of PaMIC60 and PaTAZ1 Results in Tafazzin-Independent CL 72:X Formation

**Figure 5:**
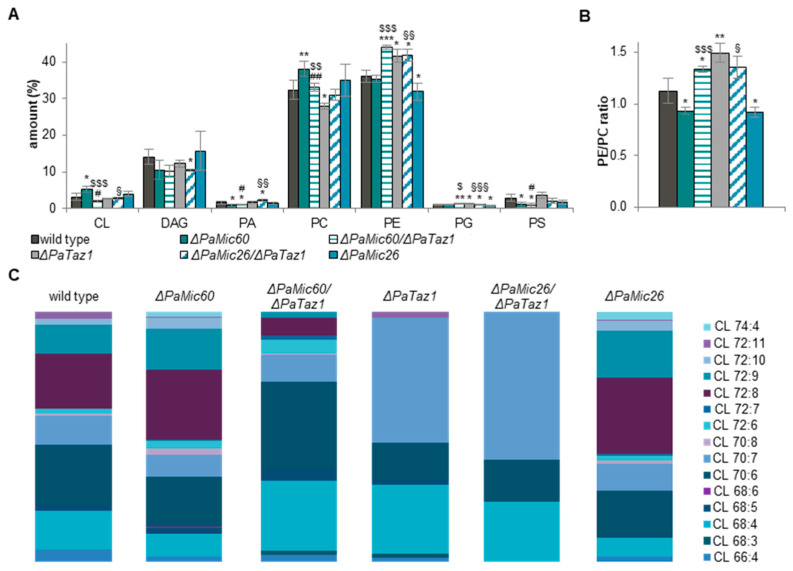
Simultaneous ablation of PaMIC60 and PaTAZ1 results in PaTAZ1-independent CL 72:X formation. (**A**) Phospholipid composition of the CDP-DAG pathway using mitochondrial protein extracts of *P. anserina* wild type (*n* = 5), Δ*PaMic60* (*n* = 5), Δ*PaMic60*/Δ*PaTaz1* (*n* = 3), Δ*PaTaz1* (*n* = 3), Δ*PaMic26*/Δ*PaTaz1* (*n* = 3), and Δ*PaMic26* (*n* = 5). Data represent mean ± SD. Exact values are shown in Supplementary Materials (Table S1). CL: cardiolipin; DAG: diacylglycerol; PA: phosphatidic acid; PC: phosphatidylcholine; PE: phosphatidylethanolamine; PG: phosphatidylglycerol; PS: phosphatidylserine. (**B**) PE/PC ratio of cultures from (**A**). Data represent mean ± SD. (**C**) Graphical illustration of different CL species in wild type, Δ*PaMic60*, Δ*PaMic60*/Δ*PaTaz1*, Δ*PaTaz1*, Δ*PaMic26*/Δ*PaTaz1*, and Δ*PaMic26* according to total length of all four acyl chains (66–74) and total degree of unsaturation (3–11). The most abundant CL species are represented. Exact values are shown in Supplementary Materials (Table S2). Significant differences to wild type: * *p* < 0.05, ** *p* < 0.01, *** *p* < 0.001; ^#^ significant differences to Δ*PaTaz1*: ^#^ *p* < 0.05, ^##^ *p* < 0.01; significant differences to Δ*PaMic60*: ^$^ *p* < 0.05, ^$$^ *p* < 0.01, ^$$$^ *p* < 0.001; significant differences to Δ*PaMic26*: ^§^ *p* < 0.05, ^§§^ *p* < 0.01, ^§§§^ *p* < 0.001.

In order to assess MICOS- and PaTAZ1-dependent changes in mitochondrial phospholipid metabolism, we next investigated the phospholipid profile in Δ*PaMic60*/Δ*PaTaz1* and Δ*PaMic26*/Δ*PaTaz1* double deletion mutants. Exact values of all analyzed phospholipids are shown in Supplementary Materials (Table S1). The most prominent changes were observed with phospholipids of the CDP-DAG pathway (Figure 5A). The amounts of PE and PG were significantly increased in Δ*PaMic60*/Δ*PaTaz1* compared to the single *PaMic60* deletion mutant. Furthermore, in addition to decreased PC abundance, we observed a significant reduction of CL level by approximately 60% (2.1% vs. 5.3%). Likewise, the simultaneous loss of PaMIC26 and PaTAZ1 led to reduced PA, PE, and PG levels compared to the *PaMic26* deletion mutant. There was also a slight, non-significant reduction in PC amount and an enhanced, significant decrease of CL level by approximately 30% (2.8% vs. 4.0%) observed. Thus, changes in phospholipid composition due to the ablation of PaTAZ1 were similar in the two subcomplex mutants. In both Δ*PaMic60*/Δ*PaTaz1* and Δ*PaMic26*/Δ*PaTaz1*, the altered amounts of PE and PC resulted in a significant increase of PE/PC ratio from 0.9 to 1.3 compared to the corresponding single deletion mutant (Figure 5B).

Surprisingly, there were pronounced differences in the CL acyl chain composition of the Δ*PaMic60*/Δ*PaTaz1* and Δ*PaMic26*/Δ*PaTaz1* double deletion mutants (Figure 5C and Figure S3). Δ*PaMic26*/Δ*PaTaz1* only exhibited the species CL 68:4, CL 70:7, and CL 70:7 and thereby hardly differed from the single *PaTaz1* deletion mutant. In contrast to Δ*PaTaz1*, the Δ*PaMic26*/Δ*PaTaz1* double mutant still had an extended lifespan (Figure 3E,F), despite the lack of long-chained CL species. Based on our previously described data, we concluded that longevity of the Mic10-subcomplex mutant Δ*PaMic26* is the result of ROS-dependent mitohormesis [21] and does not depend on phospholipid metabolism. This conclusion is supported by the finding that loss of long-chained CL species in the mutant background only partially abrogates lifespan extension. A completely different situation was observed in the Δ*PaMic60*/Δ*PaTaz1* mutant. Here, we observed a marked change in the distribution of CL species in Δ*PaMic60*/Δ*PaTaz1* compared to the Δ*PaMic60* and Δ*PaTaz1* single mutants. The most common species were CL 70:6 and CL 68:4. Further, the CL 70:7 species was not dominating, and therefore the profile was more similar to Δ*PaMic60* and wild type than to the Δ*PaTaz1* mutant. Interestingly, small amounts of CL 72:X species were found in Δ*PaMic60*/Δ*PaTaz1*, although this was not expected from the characteristics of the single *PaTaz1* deletion mutant. The most common long-chained CL species in the Δ*PaMic60*/Δ*PaTaz1* double mutant was CL 72:8, with approximately 7%. However, the amount of CL 72:8 was still significantly reduced by 75% compared to the single *PaMic60* deletion mutant and by approximately 70% compared to the wild type. This was accompanied by abolished longevity in the Δ*PaMic60*/Δ*PaTaz1* double mutant (Figure 3B,C). Although the CL 72:8 level was lower than in the wild type, the lifespans did not differ. Obviously, concomitant ablation of PaMIC60 and PaTAZ1 resulted in a more wild-type-like CL acyl chain composition and PaTAZ1-independent CL 72:X formation.

One possible explanation for the PaTAZ1-independent formation of CL 72:X could be an increased availability of 18-chain acyl residues, possibly caused by the upregulation of corresponding enzymes in fatty acid synthesis. Data from the shotgun lipidomic analysis provided a first indication of this possibility. Loss of PaMIC60 led to a significant increase of approximately 15% in phospholipids with a total chain length of 36 C atoms (2 × 18) compared to the wild type (Figure S3B). However, transcript analyses of genes coding for two enzymes (PaELO1 and PaOLE1) involved in C18 fatty acid biosynthesis argued against this hypothesis (data not shown). Another possible explanation for the formation of CL 72:X species is based on the observation that in addition to the transacylase TAZ1, other enzymes may be implicated in CL remodeling. In mammals, the acyl-CoA:lysocardiolipin acyltransferase 1 (ALCAT1) is located in mitochondria-associated membranes of the ER and shows predominant activity and selectivity towards linoleic acid (18:2) and oleic acid (18:1) [50,51]. Another enzyme in humans is the monolysocardiolipin acyltransferase 1 (MLCLAT1), which is a splice variant of the trifunctional enzyme subunit alpha and is localized in mitochondria [52,53]. In Barth syndrome lymphoblasts, overexpression of *Mlclat1* increases the amount of CL as well as the incorporation of linoleic acid into CL [54]. To determine if these alternatives also play a role in *P. anserina*, we searched for corresponding homologs of human ALCAT1 (UniProt: Q6UWP7) and human MLCLAT1 (UniProt: P40939). In a protein BLAST search [55], we identified Pa_2_4320 (UniProt: B2B5D3, E-value 9 × 10^−24^) as a homolog to human ALCAT1 and Pa_2_4980 (UniProt: B2B5L0, E-value 2 × 10^−33^) as a homolog to human MLCLAT1. We speculate that in the absence of Mic60-subcomplex and PaTAZ1, these alternative acyltransferases resume CL remodeling. Taken together, our data show that specifically the simultaneous loss of PaMIC60 and PaTAZ1 leads to an alternative mechanism for the formation of CL 72:X, which emphasizes the importance of CL remodeling in Mic60-subcomplex mutants.

### 2.5. Linoleic Acid Re-Establishes Longevity of ΔPaMic60/ΔPaTaz1

The simultaneous ablation of PaMIC60 and PaTAZ1 significantly abrogates the lifespan extension observed in a single *PaMic60* deletion mutant. We speculate that the reduced level of CL 72:X in the Δ*PaMic60*/Δ*PaTaz1* double mutant compared to Δ*PaMic60* is responsible for this effect. Moreover, specifically CL 72:8 consisting of four linoleic acid residues may play an important role in lifespan extension of mutants lacking the Mic60-subcomplex. To test this idea, we first determined the influence of linoleic acid added to the growth medium, which enhances the incorporation of this fatty acid in newly synthesizes phospholipids, thereby increasing the amounts of CL 72:8 [40]. If this assumption is correct, the lifespan of the Δ*PaMic60*/Δ*PaTaz1* double mutant should be extended by treatment with linoleic acid. First, we analyzed linoleic acid uptake in *P. anserina*. In general, after absorption from the environment, fatty acids are transported or stored in so-called lipid droplets [56]. To find a suitable concentration for further experiments, we performed growth tests using the wild type on medium containing different concentrations of linoleic acid (0.16 mM, 0.8 mM, 1.6 mM) (Figure S4A). All concentrations reduced the growth rate of the wild type. We decided to use 0.8 mM linoleic acid, a concentration at which the growth rate of the wild type was clearly impaired. A beneficial effect of linoleic acid on lifespan of Δ*PaMic60*/Δ*PaTaz1* would strongly argue for a critical role of this specific fatty acid in this mutant. To examine the entry of linoleic acid, the wild type was cultured on standard medium or on standard medium containing linoleic acid (0.8 mM). One day later, we stained the strains with the lipid droplet dye LipidSpot™ and performed fluorescence microscopy. Lipid droplets were only detected in the wild type grown on the linoleic acid-containing medium (Figure S4B), indicating the uptake of linoleic acid from the medium and its storage/transport in lipid droplets.

**Figure 6:**
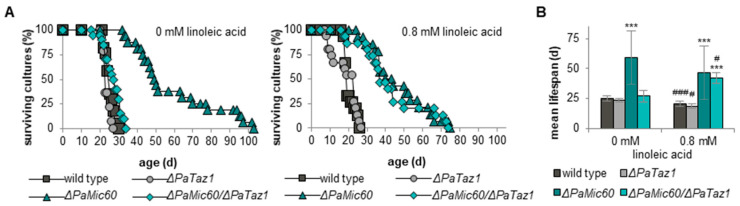
Linoleic acid leads to lifespan extension of Δ*PaMic60*/Δ*PaTaz1*. (**A**) Survival curves of *P. anserina* wild type (*n* = 17), Δ*PaTaz1* (*n* = 14), Δ*PaMic60* (*n* = 16), and Δ*PaMic60*/Δ*PaTaz1* (*n* = 20) grown on M2 medium without linoleic acid and *P. anserina* wild type (*n* = 15), Δ*PaTaz1* (*n* = 15), Δ*PaMic60* (*n* = 14), and Δ*PaMic60*/Δ*PaTaz1* (*n* = 15) grown on M2 medium with 0.8 mM linoleic acid. (**B**) Mean lifespan of cultures from (**A**). Data represent mean ± SD. Significant differences to wild type *** *p* < 0.001; significant differences to the corresponding strain without linoleic acid ^#^ *p* < 0.05, ^###^ *p* < 0.001.

Next, we investigated the effect of supplemented linoleic acid on the lifespan of *P. anserina*. Lifespan analyses of the wild type revealed a marginal reduction of the mean (20 d vs. 25 d) as well as maximal lifespan (26 d vs. 30 d) compared to the wild type grown on medium without linoleic acid (Figure 6A,B). Similar effects were also observed in single *PaTaz1* and *PaMic60* deletion mutants. Supplementation with linoleic acid did not affect maximal lifespan of the *PaTaz1* deletion mutant (27 d vs. 27 d), but we found an approximately 30% decrease in maximal lifespan of Δ*PaMic60* (75 d vs. 103 d) (Figure 6A). Additionally, the mean lifespans of the linoleic acid-treated Δ*PaTaz1* (19 d vs. 24 d) and Δ*PaMic60* (46 d vs. 59 d) mutants were slightly decreased (Figure 6B). Overall, treatment with linoleic acid led to slight negative effects in the wild type as well as *PaTaz1* and *PaMic60* single mutants. In these strains, it may be possible that excess linoleic acid is used to form other CL species, which are not beneficial for lifespan. More severe effects of high concentrations of linoleic acid have been demonstrated in other organisms. A previous study in human cells showed that linoleic acid inhibits tumor cell growth at concentrations above 0.3 mM [57]. Furthermore, treatment with conjugated linoleic acid, a derivate of linoleic acid, inhibits hyphal growth of *Candida albicans* [58].

To experimentally validate our hypothesis that specifically CL 72:8 plays an important role in lifespan extension of mutants lacking the Mic60-subcomplex, we next analyzed the lifespan of the Δ*PaMic60*/Δ*PaTaz1* double mutant grown on linoleic acid supplemented medium. Interestingly, the lifespan of Δ*PaMic60*/Δ*PaTaz1* double mutant was positively affected. We found a 55% prolonged mean lifespan (42 d vs. 27 d) and at least a 115% increased maximal lifespan (74 d vs. 34 d) compared to Δ*PaMic60*/Δ*PaTaz1* without supplemented linoleic acid, resulting in a Δ*PaMic60*-like lifespan (Figure 6A,B). Obviously, the supplementation with linoleic acid re-established longevity of the Δ*PaMic60*/Δ*PaTaz1* double mutant. All phospholipid classes might incorporate linoleic acid. However, we speculate that the additional linoleic acid finally leads to an increased abundance of CL 72:8, which is crucial in the double mutant for lifespan extension.

Our surprising results support the speculation that PaMIC60-ablation channels CL remodeling to other acyltransferases that normally do not preferably remodel CL. One possible explanation might be that the Mic60-subcomplex is part of a CL synthesis scaffold containing PaCRD1 and PaTAZ1. Studies in human cells demonstrated an interaction between MIC60 and components of the cardiolipin synthesis machinery [22], which supports our hypothesis. Since we found no CL 72:X species in the Δ*PaTaz1* mutant (Figure 4C), the transacylase PaTAZ1 seems to preferentially use phospholipids with linoleic acid residues for CL remodeling. The formation of especially CL 72:8 is therefore limited by the availability of PaTAZ1 in the proposed CL synthesis scaffold. Upon ablation of PaTAZ1, other acyltransferases cannot take over the remodeling activity since they are not part of the scaffold or, alternatively, are restricted to other membrane structures, e.g., at the interface between mitochondria and ER, as has been described for ALCAT1 [50]. However, loss of the Mic60-subcomplex together with PaTAZ1 ablation seems to allow other acyltransferases to come into close contact with the CL synthesis machinery. Since MICOS ablation not only affects mitochondrial phospholipid composition but also non-mitochondrial phospholipid synthesis [30], the induction of compensatory pathways is reasonable. At this time, this idea is highly speculative and needs to be experimentally addressed in more detail.

## 3. Conclusions

In the current study, we unraveled the underlying mechanism of lifespan extension caused by the loss of the Mic60-subcomplex, thereby uncovering a substantial role of the Mic60-subcomplex in phospholipid homeostasis. This dedicated role makes longevity of the Δ*PaMic60* mutant specifically vulnerable against PaTAZ1 ablation. Here, we suggest that the Mic60-subcomplex may form the basis of a CL building block that spatially controls formation of mCL. Furthermore, our data allow us to speculate on the induction of alternative mechanisms for the formation of CL 72:X in the absence of the Mic60-subcomplex and PaTAZ1. Altogether, we demonstrate that mCL is crucial for the healthspan of mutants lacking the Mic60-subcomplex. It will be of interest to see whether this impact of phospholipid metabolism is a mechanism that is specific for *P. anserina* or whether it is conserved among organisms.

## 4. Materials and Methods

### 4.1. P. anserina Strains and Cultivation

In this study, the *P. anserina* wild-type strain ‘s’ [59], Δ*PaTaz1* [29], Δ*PaMic26*, Δ*PaMic60*, Δ*PaMic19*, and Δ*PaMic10* [21], as well as the newly generated mutants Δ*PaMic26*/Δ*PaTaz1*, Δ*PaMic60*/Δ*PaTaz1*, and Δ*PaMic60*/Δ*PaCrd1* were used. Strains were cultivated on standard cornmeal agar (BMM) at 27 °C under constant light [60]. Spores were germinated on BMM with 60 mM ammonium acetate (Merck, Darmstadt, Germany; 1116.1000) at 27 °C in the dark for two days. All strains used in this study were derived from monokaryotic ascospores [60].

### 4.2. Generation of P. anserina Double Mutants

To generate double mutants, the single mutant strains were crossed with each other. From the progeny, strains containing both mutations were selected.

### 4.3. Southern Blot Analysis

DNA isolation from *P. anserina* was performed according to the protocol from Lecellier and Silar [61]. Digestion of 500 ng DNA, gel electrophoresis, and Southern blotting were carried out by standard protocols. According to the manufacturer’s protocol for Southern blot hybridization and detection, digoxigenin-labeled hybridization probes (DIG DNA Labeling and Detection Kit, Roche Applied Science, Mannheim, Germany, 11175033910) were used. The hygromycin resistance gene (*Hph*) specific hybridization probe corresponded to the 736 bp XhoI-fragment of the plasmid pSM4 [62].

### 4.4. Lifespan Analysis

Lifespans of *P. anserina* cultures derived from monokaryotic ascospores were determined as previously described on M2 medium at 27 °C and constant light [60]. The lifespan was defined as the time period in days (d) until growth stopped.

### 4.5. Isolation of Mitochondria

Mitochondria of *P. anserina* cultures were isolated and purified as previously described [60]. Disruption of grown mycelia was performed in isotonic mitochondria isolation buffer in the presence of bovine serum albumin (BSA, 0.2% (*w*/*v*)) (Sigma-Aldrich, St. Louis, MO, USA, A6003). After filtration through nettle cloth, the filtrate was sedimented by centrifugation (12,000× *g*) and resuspended in mitochondria isolation buffer without BSA. Separation of intact mitochondria from disrupted mitochondrial fractions was performed by ultracentrifugation (100,000× *g*) using a sucrose gradient (20–50% sucrose in H_2_O (*w*/*v*)). Intact mitochondria were collected, sedimented (15,000× *g*), resuspended in mitochondria isolation buffer, and stored at −80 °C.

### 4.6. Western Blot Analysis

Western blot analysis was performed with 50 µg mitochondria as previously described [19]. The primary antibody anti-PaCRD1 (rabbit, dilution 1:5000, peptide: [H]-SKKEKEVVVEEEEGKKKEL-[OH], Davids Biotechnologie GmbH, Regensburg, Germany) was used. As secondary antibodies, IRDye^®^ 680RD anti-rabbit (goat, 1:15,000 dilution, LI-COR Biosciences, Bad Homburg, Germany, 926-68071) and IRDye^®^ 800CW anti-rabbit (goat, 1:15,000 dilution, LI-COR Biosciences, Bad Homburg, Germany, 926-32211) were used. The Odyssey^®^ Fc imaging system (LI-COR Biosciences, Bad Homburg, Germany) was used for detection. Densitometric quantification was performed with the manufacturer’s software Image Studio Lite (Version 5.2).

### 4.7. Lipidomic Analysis

Mass spectrometry-based lipid analysis was performed by Lipotype GmbH (Dresden, Germany) as described [63,64]. Lipids were extracted using a two-step chloroform/methanol procedure [63]. Samples were spiked with internal lipid standard mixture containing CDP-DAG 17:0/18:1, cardiolipin 14:0/14:0/14:0/14:0 (CL), ceramide 18:1;2/17:0 (Cer), diacylglycerol 17:0/17:0 (DAG), lyso-phosphatidate 17:0 (LPA), lyso-phosphatidyl-choline 12:0 (LPC), lyso-phosphatidylethanolamine 17:1 (LPE), lyso-phosphatidylinositol 17:1 (LPI), lyso-phosphatidylserine 17:1 (LPS), phosphatidate 17:0/14:1 (PA), phosphatidylcholine 17:0/14:1 (PC), phosphatidylethanolamine 17:0/14:1 (PE), phosphatidylglycerol 17:0/14:1 (PG), phosphatidylinositol 17:0/14:1 (PI), phosphatidylserine 17:0/14:1 (PS), ergosterol ester 13:0 (EE), triacylglycerol 17:0/17:0/17:0 (TAG), inositolphosphorylceramide 44:0;2 (IPC), mannosyl-inositolphosphorylceramide 44:0;2 (MIPC), and mannosyl-di-(inositolphosphoryl)ceramide 44:0;2 (M(IP)_2_C). After extraction, the organic phase was transferred to an infusion plate and dried in a speed vacuum concentrator. In the first step, dry extract was re-suspended in 7.5 mM ammonium acetate in chloroform/methanol/propanol (1:2:4, *v*:*v*:*v*); in the second step, dry extract was re-suspended in 33% ethanol solution of methylamine in chloroform/methanol (0.003:5:1; *v*:*v*:*v*). All liquid handling steps were performed using the Hamilton Robotics STARlet robotic platform (Reno, NV, USA) with the Anti Droplet Control feature for organic solvents pipetting.

Samples were analyzed by direct infusion on a QExactive mass spectrometer (Thermo Scientific, Waltham, MA, USA) equipped with a TriVersa NanoMate ion source (Advion Biosciences, Ithaca, NY, USA). Samples were analyzed in both positive and negative ion modes with a resolution of R*_m_*_/*z*=200_ = 280,000 for MS and R*_m_*_/*z*=200_ = 17,500 for MSMS experiments, in a single acquisition. MSMS was triggered by an inclusion list encompassing corresponding MS mass ranges scanned in 1 Da increments [65]. Both MS and MSMS data were combined to monitor EE, DAG, and TAG ions as ammonium adducts; PC as an acetate adduct; and CL, PA, PE, PG, PI, and PS as deprotonated anions. MS only was used to monitor LPA, LPE, LPI, LPS, IPC, MIPC, and M(IP)_2_C as deprotonated anions; and Cer and LPC as acetate adducts.

Data were analyzed with in-house developed lipid identification software based on LipidXplorer [66,67]. Data post-processing and normalization were performed using an in-house developed data management system. Only lipid identifications with a signal-to-noise ratio >5, and a signal intensity 5-fold higher than in corresponding blank samples were considered for further data analysis.

### 4.8. Thin Layer Chromatography

Phospholipid extraction from isolated mitochondria and thin layer chromatography were performed as previously described [29].

### 4.9. Fluorescence Microscopy

Strains were grown on glass slides with a central depression containing 130 µL M2 medium or linoleic acid-containing M2 medium (0.8 mM) for one day under standard conditions. For the staining of lipid droplets, grown mycelium was incubated with LipidSpot™ 488 (Biotium, Fremont, CA, USA; 70065) for 15 min. Fluorescence microscopic analyses were performed with a fluorescence microscope (DM LB/11888011, Leica, Wetzlar, Germany) and a DFC7000 T camera; image processing was conducted with the corresponding LAS X software from Leica (Leica Microsystems GmbH, Wetzlar, Germany).

### 4.10. Statistical Analysis

Significances between different lifespans were statistically analyzed with the IBM SPSS statistics 19 software package (IBM, Armonk, NY, USA) by generating Kaplan-Meier survival estimates. We used three independent statistical tests (Breslow (generalized Wilcoxon), log-rank (Mantel-Cox), and the Tarone-Ware) with a pairwise comparison. All other statistical significances were performed by the two-tailed Student’s *t*-test. The respective samples were compared with the appropriate wild type sample. For statistical significance, the minimum threshold was set at *p* < 0.05. * *p* < 0.05; ** *p* < 0.01; and *** *p* < 0.001.

## Data Availability

Original data are available upon reasonable request from the corresponding author.

## Supplementary Materials

The following supporting information can be downloaded at: https://www.mdpi.com/article/10.3390/ijms23094741/s1.
