# Supplementary material for: Lifespan Extension of Podospora anserina Mic60-Subcomplex Mutants Depends on Cardiolipin Remodeling
Source: Int J Mol Sci. 2022 Apr 25;23(9):4741. doi: 10.3390/ijms23094741 (PMC9099538; doi:10.3390/ijms23094741)
Supplement: Supplementary file 1 [file ijms-23-04741-s001.zip › ijms-1670239-Supplementary.pdf]

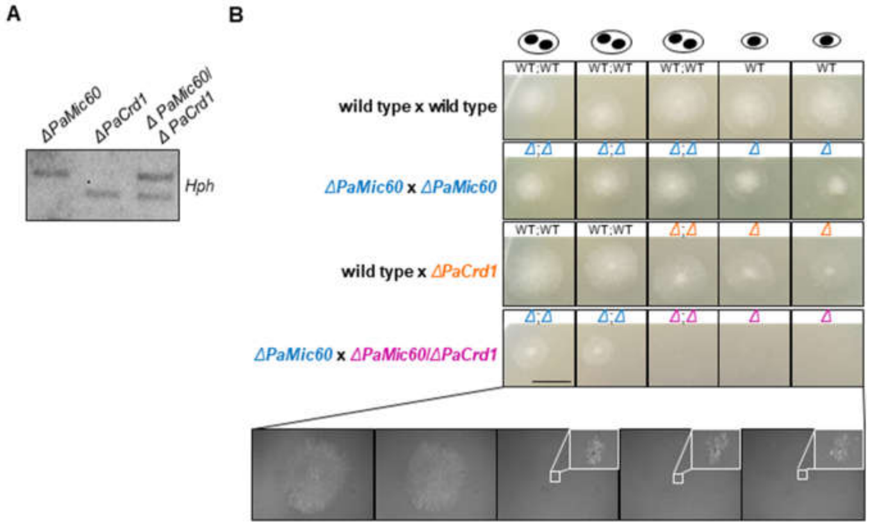

Figure S1: Southern blot verification and germination analysis of *ΔPaMic60/ΔPaCrd1*. (**A**) Southern blot analysis with EcoRV-digested DNA to verify the newly generated double mutant *ΔPaMic60/ΔPaCrd1* by the use of the hygromycin (*Hph*) resistance gene. (**B**) Comparison of germinated spores of crossing two wild-type strains (WT, black) or two *ΔPaMic60* strains (blue), of a cross of *ΔPaCrd1* (orange) with the wild type, and of a cross of *ΔPaMic60/ΔPaCrd1* (violet) with *ΔPaMic60*. Ascospores were germinated on BMM with 60 mM ammonium acetate for 2 days at 27 °C in the dark. Normally, *P. anserina* forms asci with four spores each of them containing two different nuclei giving rise to heterokaryotic mycelia. For experimental purposes monokaryotic spores are preferred, which rarely are present in so-called "irregular asci". Here, irregular asci with five spores, consisting of 3 dikaryotic and 2 monokaryotic spores were used [60]. 'WT' or '*Δ*' in the respective colors indicate the genotype of each nucleus in a spore. Isolated spores, either di- or monokaryotic, of the *ΔPaMic60/ΔPaCrd1* double mutant germinate poorly due to a germination defect. The last horizontal row shows a magnification of the spores of the *ΔPaMic60/ΔPaCrd1* x *ΔPaMic60* cross. The inlay presents an additional magnification to visualize the spores with *ΔPaMic60/ΔPaCrd1* genotype. After initial formation of germination tubes, growth of the double mutant completely stops. Scale bar = 1 cm.

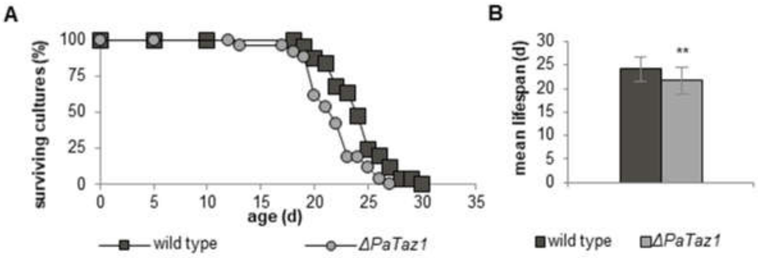

Figure S2: Loss of PaTAZ1 affects lifespan of *P. anserina*. (**A**) Survival curves of wild type (n = 25) and *ΔPaTaz1* (n = 26) grown on M2 medium. (**B**) Mean lifespan of cultures from (A). Data represent mean ± SD. ** $p < 0.01$.

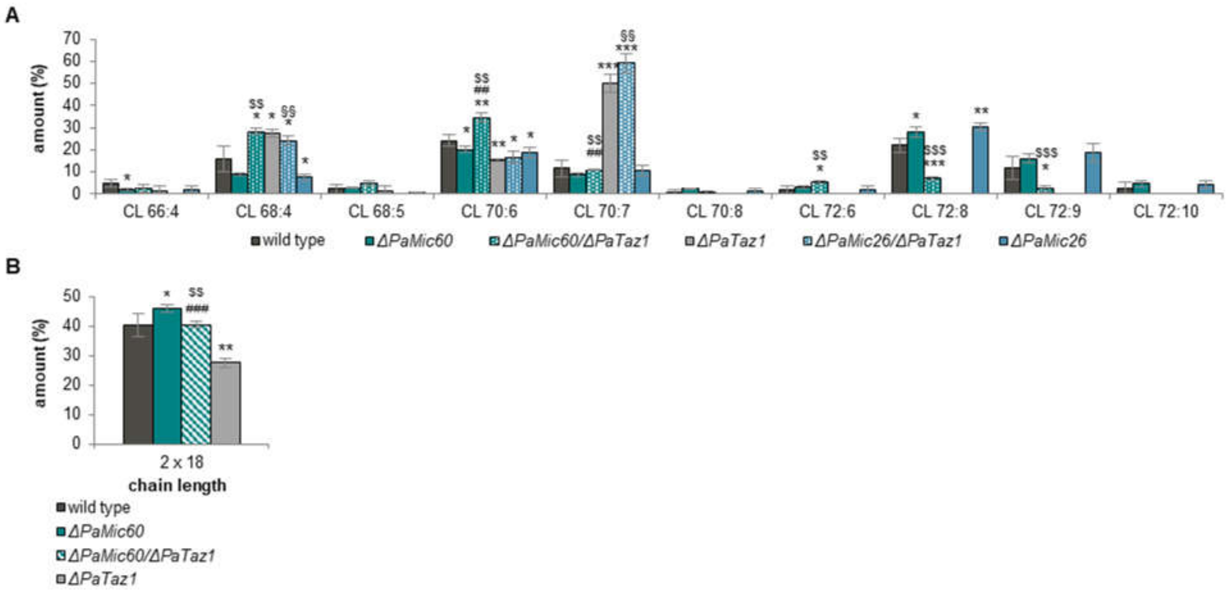

Figure S3: Ablation of PaMIC60 and PaTAZ1 alters CL species and amount of 18-chained acyl residues incorporated into membrane lipids. (**A**) Different CL species in wild type, *ΔPaMic60*, *ΔPaMic60*/*ΔPaTaz1*, *ΔPaTaz1*, *ΔPaMic26*/*ΔPaTaz1*, and *ΔPaMic26* according to total length of all four acyl chains (66-72) and total degree of unsaturation (4-10). The most abundant CL species are represented. (**B**) Alterations in the amount of 18-chained acyl residues across all identified lipid classes in wild type, *ΔPaMic60*, *ΔPaMic60*/*ΔPaTaz1*, and *ΔPaTaz1*. * Significant differences to wild type, * $p < 0.05$ ** $p < 0.01$ *** $p < 0.001$; # significant differences to *ΔPaTaz1*, ## $p < 0.01$; $ significant differences to *ΔPaMic60*, $$ $p < 0.01$, $$$ $p < 0.001$; § significant differences to *ΔPaMic26*, § $p < 0.05$, §§ $p < 0.01$.

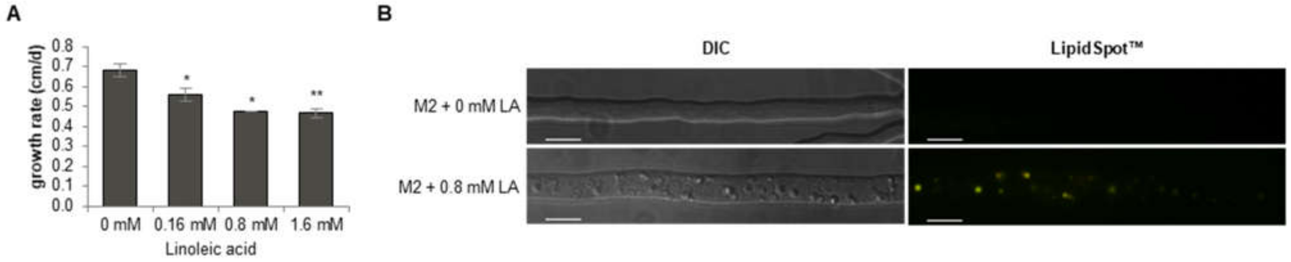

Figure S4: Growth tests and appearance of lipid droplets after supplementation with linoleic acid (LA). (**A**) Growth tests of the wild type (n = 3) grown on M2 medium with or without different concentrations of LA. Data represent mean ± SD. * $p < 0.05$, ** $p < 0.01$. (**B**) Staining of wild type grown on M2 medium with or without 0.8 mM LA for one day. Visualization was performed using LipidSpot™. Scale bar = 10 µm.

Table S4: Overview of lifespan and growth rate of wild type, *ΔPaTaz1*, *ΔPaMic60*, and *ΔPaMic60/ΔPaTaz1* mutants. The *p*-values were determined with SPSS (IBM; SPSS Statistics, New York, USA) with three different tests. Indicated are the *p*-values of the lifespan curves in comparison to the wild type. "*p*-value 1" = Log Rank (Mantel-Cox); "*p*-value 2" = Breslow (Generalized Wilcoxon); "*p*-value 3" = Tarone-Ware. Moreover, *p*-values of the lifespan curves compared to *ΔPaMic60*. "*p*-value 4" = Log Rank (Mantel-Cox); "*p*-value 5" = Breslow (Generalized Wilcoxon); "*p*-value 6" = Tarone-Ware. And *p*-values of the lifespan curves compared to *ΔPaTaz1*. "*p*-value 7" = Log Rank (Mantel-Cox); "*p*-value 8" = Breslow (Generalized Wilcoxon); "*p*-value 9" = Tarone-Ware.

	**wild type**	***ΔPaTaz1***	***ΔPaMic60***	***ΔPaMic60/ ΔPaTaz1***
**mean lifespan (d) ± SD**	24 ± 2.7	22 ± 2.9	54 ± 20.2	26 ± 6.2
**maximum lifespan (d)**	30	27	103	39
p-value 1	/	0.0062	1.257E-13	0.0107
p-value 2	/	0.0062	1.854E-11	0.1451
p-value 3	/	0.0059	1.632E-12	0.0476
p-value 4	/	1.001E-13	/	1.772E-13
p-value 5	/	1.742E-11	/	3.726E-11
p-value 6	/	1.398E-12	/	2.511E-12
p-value 7	/	/	1.001E-13	9.042E-05
p-value 8	/	/	1.742E-11	2.264E-03
p-value 9	/	/	1.398E-12	5.147E-04
**growth rate (cm/d) ± SD**	0.66 ± 0.03	0.64 ± 0.02	0.64 ± 0.02	0.61 ± 0.02
**growth distance (cm) ± SD**	14.0 ± 1.5	11.9 ± 1.6	32.6 ± 14.0	14.2 ± 3.7
biological replicates	25	26	24	32

Table S5: Overview of lifespan and growth rate of wild type, *ΔPaTaz1*, *ΔPaMic26*, and *ΔPaMic26/ΔPaTaz1* mutants. The *p*-values were determined with SPSS (IBM; SPSS Statistics, New York, USA) with three different tests. Indicated are the *p*-values of the lifespan curves in comparison to the wild type. "*p*-value 1" = Log Rank (Mantel-Cox); "*p*-value 2" = Breslow (Generalized Wilcoxon); "*p*-value 3" = Tarone-Ware. Moreover, *p*-values of the lifespan curves compared to *ΔPaMic26*. "*p*-value 4" = Log Rank (Mantel-Cox); "*p*-value 5" = Breslow (Generalized Wilcoxon); "*p*-value 6" = Tarone-Ware. And *p*-values of the lifespan curves compared to *ΔPaTaz1*. "*p*-value 7" = Log Rank (Mantel-Cox); "*p*-value 8" = Breslow (Generalized Wilcoxon); "*p*-value 9" = Tarone-Ware.

	**wild type**	***ΔPaTaz1***	***ΔPaMic26***	***ΔPaMic26/ ΔPaTaz1***
**mean lifespan (d)**<br>**± SD**	24<br>± 4.1	21<br>± 3.9	53<br>± 13.2	34<br>± 11.8
**maximum lifespan (d)**	32	29	85	64
p-value 1	/	0.0032	4.383E-20	1.025E-07
p-value 2	/	0.0093	5.545E-18	2.821E-06
p-value 3	/	0.0050	5.039E-19	5.277E-07
p-value 4	/	3.320E-21	/	5.246E-10
p-value 5	/	9.761E-19	/	4.513E-10
p-value 6	/	5.843E-20	/	3.078E-10
p-value 7	/	/	3.320E-21	1.765E-12
p-value 8	/	/	9.761E-19	3.807E-10
p-value 9	/	/	5.843E-20	2.750E-11
**growth rate (cm/d)**<br>**± SD**	0.66<br>± 0.02	0.63<br>± 0.03	0.67<br>± 0.06	0.62<br>± 0.04
**growth distance (cm)**<br>**± SD**	13.6<br>± 2.8	11.8<br>± 2.6	33.1<br>± 8.9	19.3<br>± 7.2
biological replicates	24	27	43	50

Table S6: Overview of lifespan and growth rate of wild type, *ΔPaTaz1*, *ΔPaMic60*, and *ΔPaMic60*/*ΔPaTaz1* mutants with and without linoleic acid. The *p*-values were determined with SPSS (IBM; SPSS Statistics, New York, USA) with three different tests. Indicated are the *p*-values of the lifespan curves in comparison to the wild type without linoleic acid. "*p*-value 1" = Log Rank (Mantel-Cox); "*p*-value 2" = Breslow (Generalized Wilcoxon); "*p*-value 3" = Tarone-Ware. Additionally, *p*-values of the lifespan curves compared to the corresponding deletion mutant without linoleic acid are given. "*p*-value 4" = Log Rank (Mantel-Cox); "*p*-value 5" = Breslow (Generalized Wilcoxon); "*p*-value 6" = Tarone-Ware.

	**wild type**	***ΔPaTaz1***	***ΔPaMic60***	***ΔPaMic60/ ΔPaTaz1***	**wild type**	***ΔPaTaz1***	***ΔPaMic60***	***ΔPaMic60/ ΔPaTaz1***
	**0 mM linoleic acid**				**0.8 mM linoleic acid**			
**mean lifespan (d)**<br>**± SD**	25<br>± 2.3	24<br>± 1.7	59<br>± 22.1	27<br>± 4.6	20<br>± 3.0	19<br>± 6.7	46<br>± 14.5	42<br>± 16.7
**maximum lifespan (d)**	30	27	103	34	26	27	75	74
p-value 1	/	0.0682	1.702E-09	0.0228	6.692E-04	0.0051	2.037E-07	5.291E-06
p-value 2	/	0.0950	4.898E-08	0.1259	1.907E-04	0.0032	3.394E-06	2.021E-04
p-value 3	/	0.0816	9.333E-09	0.0584	3.132E-04	0.0037	8.503E-07	3.514E-05
p-value 4	/	/	/	/	/	0.2192	0.0966	3.695E-05
p-value 5	/	/	/	/	/	0.1014	0.1413	7.071E-04
p-value 6	/	/	/	/	/	0.1418	0.1264	1.684E-04
**growth rate (cm/d)**<br>**± SD**	0.65<br>± 0.02	0.64<br>± 0.02	0.63<br>± 0.02	0.62<br>± 0.02	0.46<br>± 0.02	0.48<br>± 0.02	0.44<br>± 0.02	0.41<br>± 0.02
**growth distance (cm)**<br>**± SD**	14.4<br>± 1.1	12.9<br>± 0.8	36.2<br>± 15.5	14.9<br>± 2.8	7.7<br>± 1.2	7.5<br>± 3.2	19.1<br>± 6.4	16.5<br>± 6.7
biological replicates	17	14	16	20	15	15	14	15
